# Supplementary material for: BEEM-Static: Accurate inference of ecological interactions from cross-sectional microbiome data
Source: PLoS Comput Biol. 2021 Sep 8;17(9):e1009343. doi: 10.1371/journal.pcbi.1009343 (PMC8452072; doi:10.1371/journal.pcbi.1009343)

Derivation of the expectation-maximization algorithm

Given the regression model:

$$\mathrm{Y}_{ik} = \frac{a_i}{m_k} + \sum_{j=1, j\neq i}^{p} b_{ij}\mathrm{X}_{jk} + \epsilon, \epsilon \sim \mathrm{Normal}(0, \delta^2),$$

where $\mathrm{Y}_{ik} = \tilde{x}_{ik}$ and $\mathrm{X}_{jk} = \tilde{x}_{jk}$ $(j \neq i)$ are relative abundances in sample k, we can derive the following log likelihood function:

$$Q\left(\boldsymbol{a}, \boldsymbol{b} \middle| \boldsymbol{a}^{(T-1)}, \boldsymbol{b}^{(T-1)}\right) = E_{\boldsymbol{M}|\boldsymbol{a}^{(T-1)}, \boldsymbol{b}^{(T-1)}, \boldsymbol{X}, \boldsymbol{Y}}[\mathcal{L}(\boldsymbol{a}, \boldsymbol{b}; \boldsymbol{X}, \boldsymbol{Y}, \boldsymbol{M})]$$
$$= \int \mathcal{L}(\boldsymbol{a}, \boldsymbol{b}; \boldsymbol{X}, \boldsymbol{Y}, \boldsymbol{M}) \delta(\boldsymbol{M} - \boldsymbol{m}) d\boldsymbol{M} = \mathcal{L}(\boldsymbol{a}, \boldsymbol{b}; \boldsymbol{X}, \boldsymbol{Y}, \boldsymbol{m})$$
$$= \prod_k \prod_i \frac{1}{\sqrt{2\pi\sigma^2}} e^{-\frac{\left(Y - \frac{a_i}{m_k} - \sum_{j=1, j\neq i}^{p} b_{ij}\mathrm{X}_{jk}\right)^2}{2\sigma^2}},$$

where $\boldsymbol{a}$ and $\boldsymbol{b}$ are model parameters and $\delta(\boldsymbol{M})$ is a Dirac delta function for the biomass values and $\mathcal{L}(\boldsymbol{a}, \boldsymbol{b}; \boldsymbol{X}, \boldsymbol{Y}, \boldsymbol{m})$ is the likelihood function with respect to $\boldsymbol{a}$ and $\boldsymbol{b}$ for the regression problem. The parameters can then be solved using the following EM algorithm:

E-step: The re-parameterized gLV equation can be written as:

$$a_i + m \sum_{j=1}^{p} b_{ij} \tilde{x}_j = 0, b_{ij} = 1.$$

Then the biomass value can be derived by re-arranging the terms:

$$m = -\frac{a_i}{\sum_{j=1}^{p} b_{ij} \tilde{x}_j}.$$

In iteration T, with $\hat{a}_i^{(T-1)}$ and $\hat{b}_{ij}^{(T-1)}$ estimated from the previous iteration, the biomass $\widehat{m}_k^{(T)}$ for each T can be computed as follows:

$$\widehat{m}_k^{(T)} = E\left[-\frac{\hat{a}_i^{(T-1)}}{\sum_{j=1}^{p} \hat{b}_{ij}^{(T-1)} \mathrm{X}_j}\right].$$

In practice, we compute the median value instead of the mean to avoid the effect of outliers.

M-step: the new model parameters $\hat{a}_i^{(T)}$ and $\hat{b}_{ij}^{(T)}$ are then solved as the following regression problem:

$$< \widehat{\boldsymbol{a}}^{(T)}, \widehat{\boldsymbol{b}}^{(T)} > = \underset{\boldsymbol{a}, \boldsymbol{b}}{\operatorname{argmax}} \log \left(Q\left(\boldsymbol{a}, \boldsymbol{b} \middle| \boldsymbol{a}^{(T-1)}, \boldsymbol{b}^{(T-1)}\right)\right) = \underset{\boldsymbol{a}, \boldsymbol{b}}{\operatorname{argmax}} \log \left(L\left(\boldsymbol{a}, \boldsymbol{b}; \boldsymbol{X}, \boldsymbol{Y}, \widehat{\boldsymbol{m}}^{(T)}\right)\right).$$

Note that, as expected, scaling factors ($\boldsymbol{m}$) and carrying capacities ($\boldsymbol{a}$) from BEEM-Static cannot recapitulate the absolute scale (infinitely many solutions by scaling $\boldsymbol{m}$ and $\boldsymbol{a}$ with an arbitrary constant), and so their estimates were scaled to the same median value as the ground

truth for calculating relative errors. In practice, the true scale can be recovered by measuring the biomass for a single sample accurately.

Convergence of BEEM-Static on simulated and real datasets

We observed that the EM algorithm used by BEEM-Static converged well on both simulated (**A**; sample dataset) and real datasets (**B**).

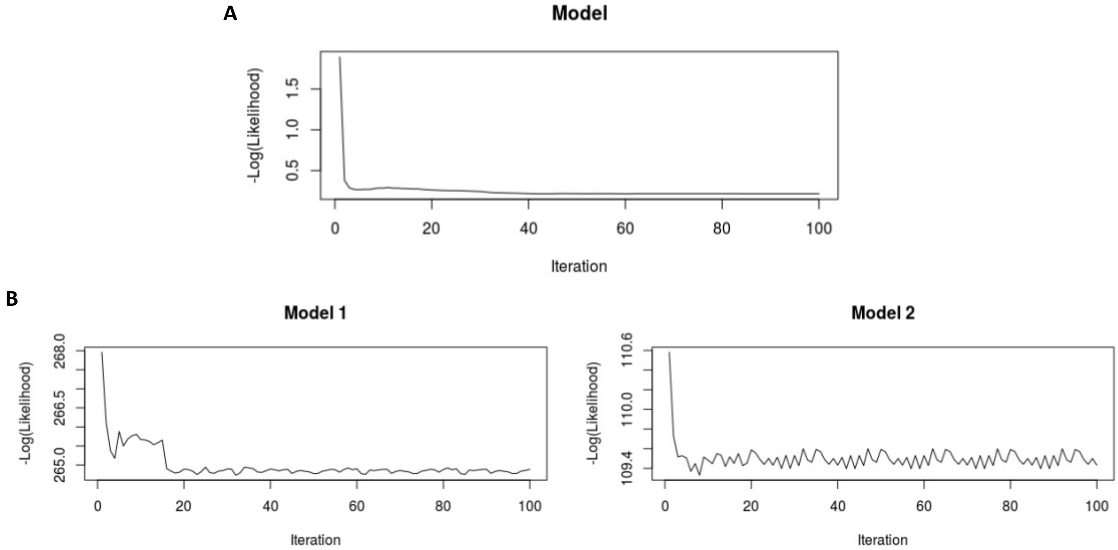

Supplement: S1 Text — (PDF) [file pcbi.1009343.s009.pdf]
