# Supplementary material for: BEEM-Static: Accurate inference of ecological interactions from cross-sectional microbiome data
Source: PLoS Comput Biol. 2021 Sep 8;17(9):e1009343. doi: 10.1371/journal.pcbi.1009343 (PMC8452072; doi:10.1371/journal.pcbi.1009343)
Supplement: Attachment — Submitted filename: response letter.pdf [file pcbi.1009343.s010.pdf]

We thank both reviewers for their summaries and constructive comments on our manuscript. We provide point-by-point responses to address the reviewers' concerns below and have edited the manuscript according to these suggestions.

Reviewer #1:

Li et al. developed a tool called "BEEM-Static" to infer microbial interactions from cross-sectional metagenomic data. These interactions are inferred using a generalized Lotka-Volterra model (gLVM). The authors highlight that in contrast to correlation-based methods BEEM-Static is able to infer directionality in addition to presence of the interactions. Further, they provide estimates of the sample biomass. They benchmarked their algorithm against 10 correlation-based methods using simulated datasets, simple pairwise experimental co-culture data and a large cross-sectional metagenomic dataset. BEEM-Static was robust to violations of model assumptions (equilibrium states and model differences) in these tests. While their method is interesting and the benchmarking results show improvements over other tools, I have several concerns:

Response: We thank the reviewer for highlighting the salient features of our work.

Major concerns

1. What is the main difference to the original BEEM algorithm? The filter functions? Please add more details on that to the introduction.

Response: We apologize for this not being as clear as it should be. We list the major differences between BEEM-Static and BEEM here and have added more details to the introduction section as suggested by the reviewer.

- input data type: BEEM takes in longitudinal microbiome data as input while BEEM-Static uses cross-section microbiome data.
- assumptions: BEEM assumes that the microbiome dynamics of the longitudinal data of interest can be approximated by a gLVM. BEEM-Static additionally assumes that most of the samples are at or close to the equilibrium ("static").
- Model and filters: BEEM-static derives a special case of BEEM which does not require multiple timepoints from the same individual. In addition, to account for violations in its assumptions, it uses a filtering approach to identify and remove samples.

2. Further details about model training should be provided. For example, additional figures showing how well the model converged by the end of training.

Response: We thank the reviewer for this suggestion. We have now included a figure in the **Supplementary Text** that demonstrates that the training converges well in practice.

3. The parameter inference method used in the paper is a coordinate descent algorithm, a general form of EM-algorithm. For the EM algorithm, it is necessary to write the log-likelihood function and formulate the E-step and M-step in this framework. That way, the M-step is maximizing the lower bound of the log-likelihood function which is an essential guarantee for the model to converge. In the general coordinate descent, it is necessary to prove that the function is bounded in order to show that the algorithm will converge. This is missing currently.

Response: We thank the reviewer for pointing this out. We had originally relied on the fact that these details were provided in the BEEM manuscript, of which BEEM-static is a special case. For completeness, we have now provided the derivation of the EM algorithm for BEEM-Static in **Supplementary Text**.

4. In the large-scale metagenomic dataset, only 42 species passed the filtering. This number is very small for human gut microbial communities and a large fraction of the microbial community is not considered in many samples. I would suggest the authors provide a barplot showing the fraction of considered microbes in each sample.

Response: We thank the reviewer for this comment. The 42 species considered cover 65% of the gut microbiome on average. The filter was used to ensure that the number of parameters (which grows quadratically with the number of species, in the worst case) is conservatively within control. We have now included a plot (**Supplementary Figure 6**) as requested by the reviewer. We also added this limitation to the discussion section.

5. The authors didn't report how many samples are found invalid in the large-scale metagenomic dataset. They showed that the model still works well when up to 40% of samples are at non-equilibrium. Without the number of how many samples are predicted as "invalid" in the metagenomic dataset, it is uncertain if this criteria is met for the large metagenomic dataset.

Response: We apologize for not including this information. In total 1,477 out of 4,617 (32%) samples were filtered out. We have now added this number to the manuscript.

6. I would recommend a graphical overview of BEEM-static as one of the first figures, including input, output and potential conclusions that can be inferred from the results.

Response: We agree with the reviewer that a graphical overview would help the reader understand the idea and usage of BEEM-Static better. We have now included a figure for this (**Figure 1**).

7. I’m curious what the rationale is behind the biomass estimation. Why should it be possible to infer biomass based on relative abundances?

Response: This is a good question. For one particular sample, we can write a gLV equation for a species. If we have estimated the gLV parameters, the biomass is the only unknown variable left in the equation. We can then estimate the biomass because there are multiple such equations (i.e. multiple data points) in the sample sharing the same biomass value. This is the intuition behind the EM algorithm. In a recent work, Remien *et al* have also showed theoretically that the parameters of gLVM are identifiable with relative abundances.

Reference: https://www.biorxiv.org/content/10.1101/463372v1.full

8. Last paragraph of results section: Why do you point out fibre might play a role here? Without the context from the cohort, this seems a bit random. Please describe the real world datasets you used briefly (Healty cohort? Disease?) and why fibre is relevant.

Response: We thank the reviewer for highlighting this point. We have mentioned in the method section that the samples used in this study were from healthy adults. We have observed that the carrying capacity of *Prevotella copri* is a major contributor to the differences between the two models inferred. Many studies have previously reported the abundance of *P. copri* as being associated with fibre intake (De Filippo *et al*., 2010, Koeth *et al*., 2013, Wu *et al*., 2011, Kovatcheva-Datchary *et al*., 2015), so we believe it is a reasonable hypothesis that our observation may be a result of differences in dietary fibre intake. We have edited the text to further clarify this point.

9. Why did you expect that enterotypes play a role in this or are affected by interactions?

Response: We apologize for this confusion. We would like to clarify that we did not mean to claim that the conventionally defined enterotypes (clustering based on Bray-Curtis or other distance metrics) play a role in the two models that we learned. The expected equilibria of the two models (defined by different interactions and carrying capacities) were observed to form three different clusters, where most of the samples from cluster #1 and cluster #2 were from distinct models on the PCA plot. In contrast, the samples from model #2 were mixed with samples from model #1 on the PCoA plot, which is usually used to identify enterotypes (Arumugam *et al*., 2011,).

Minor concerns:

1. I don't see why their approach is only applicable to metagenomic data. The abundance profiles from 16S data should work equally well.

Response: We agree with the reviewer that BEEM-Static is not restricted to shotgun metagenomic data. We used metagenomic data in the paper because it has better taxonomic resolution and is therefore easier to interpret. We have now edited the text to use “microbiome” instead of “metagenomic” to avoid this confusion.

2. It would be interesting to verify the model's prediction on the equilibrium state of the metagenomic samples from the other time points. Does the next timepoint deviate from the current one? Does the second time point of the samples predicted as invalid deviate more than those predicted as valid? That would provide further confidence for the model's performance.

Response: These are interesting comments. We do not observe significant difference in the distances to the predicted equilibria between time point 1 and time point 2 (left figure below). This could be attributed to the long term stability of the gut microbiome. However, the 2nd time point of the samples predicted as invalid do deviate significantly more than those predicted as valid, as expected (right figure below).

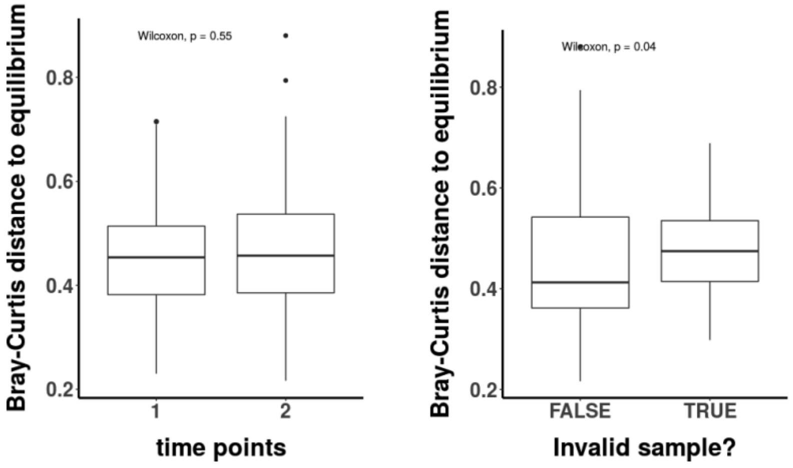

3. Given a model, there will be multiple equilibrium states (the eigenvectors of the state matrix). It would be interesting to show how these equilibrium states are distributed in the large cohort.

Response: In fact, **Figure 3C** (**Figure 4C** in the current version) is a plot for the equilibria in the dataset based on the presence-absence of species.

4. Some terms should be defined in the introduction, such as equilibrium. Examples for bacterial interactions of the different types would be helpful too (pos-pos, neg-neg, pos-neg).

Response: We thank the reviewer for this suggestion. We have now made changes in the introduction as suggested by the reviewer.

5. p. 15 (on my version): Define the abbreviation for the species involved in the interaction (DP, FP, ...).

Response: We apologize for not providing this necessary information and we have added the corresponding species names to the legend of **Figure 1** (**Figure 2** in the current version).

6. Fig 1C: Explain in the legend what those numbers in connection with the interactions mean.

Response: Numbers represent the interaction strength defined by the gLVMs or correlation coefficients inferred by BAnOCC. We have now added this information to the legend.

7. Fig 3C: The axis relative to the origin would make more sense to me. Also, I'm curious why the first PC explains so much variance? How were the clusters computed?

Response: We agree with the reviewer that the axes are usually drawn through the origin for PCA. However, we plotted the axes on the sides to avoid them going through the datapoints. The clusters were assigned visually based on the density distribution on the first two PCs.

Reviewer #2:

This manuscript introduces a new bioinformatics algorithm, "BEEM-Static", to infer ecological interactions from cross-sectional metagenomic data. The work is motivated by the current needs in obtaining deeper ecological understanding of the systems underlying observation in microbiome research in general, and in microbial metagenomics in particular. Currently popular methods for computational inference of microbial interactions are based on observed co-abundance patterns but often lacking ecological insight. This paper aims to demonstrate that ecological structure can be recovered from cross-sectional metagenomic data. The method is an EM algorithm (BEEM-Static) that utilizes a standard ecological (gLV) interaction model. Application requires sample filtering but this is justified based on the modeling assumptions: in particular, the authors show that gLV parameters can estimated directly from cross-sectional data, when it is being assumed that 1) the system is close to equilibrium, and 2) absolute abundances are known. This has considerable practical value because it helps to obtain ecologically relevant insights from dynamical systems in a setting where the lack of long and dense time series has so far limited the investigations. Comparison with relevant alternatives demonstrates remarkable improvements in the ability to detect interaction and its directionality reliably (based on AUC/ROC scores). It is also shown that the method is relatively robust to outliers and deviations from stationarity or model assumptions, so that the first assumption seems feasible. The second assumption is not generally available but the authors propose a way to estimate this computationally, and advances in measurement techniques may improve this further over time; the main contribution in this work is the method itself. An interesting demonstration of the model performance is provided by analysing community stability in a well-known public human gut microbiome data set, and the proposed model is potentially applicable to studying key aspects of community dynamics across a vast spectrum of microbiome data sets, and the results can be experimentally testable in many cases if suitable experimental time series can be collected.

The manuscript is technically sound and written in a fluent and easily understandable English. Experiments and statistical analyses have been conducted rigorously and the conclusions are supported by the data. Relevant literature is cited, and the relevant recent methods have been included in the comparisons.

All data and code are fully available but I have not tried to replicated the analysis. The associated R package seems good quality and implemented according to prevailing standards for R package development.

Response: We thank the reviewer for this detailed summary and evaluation of our work and its utility. As authors it feels great to know that others can clearly appreciate the point of our manuscript.

I only have the following minor notes:

Minor

1. The ecological models do not correspond to previously reported enterotypes (in particular Fig S6). However, enterotypes – as visualized on 2D PCoA space - are very broad community-level clusters. It is possible that there are more refined subcommunities that would be better visible on further PCoA dimensions or in a focused subcommunity analysis and these might correspond better with the previously reported enterotypes that are said to be driven by specific bacterial groups. It would be informative to include some more discussion on the possible connections. In particular, Prevotella has been shown to have bimodal/bistable abundance distribution at the population level, and if the BEEM-Static model does not match this finding, this would be somewhat unexpected and potentially indicate problems in the BEEM-Static model.

Response: We thank the reviewer for this interesting point. We plotted the next few principal coordinates (2 to 5) below and found that there was no clear separation of the two models identified by BEEM-Static. The PCA plot (**Figure 4C**) presents the expected equilibria of the samples, and so it is likely less affected by noise in abundances measured through sequencing. Nevertheless, the models predicted by BEEM-Static do capture the bistable nature of *Prevotella copri* (the two models have different carrying capacities for *P. copri*) consistent with what has been pointed out by the reviewer (**Figure 4D**).

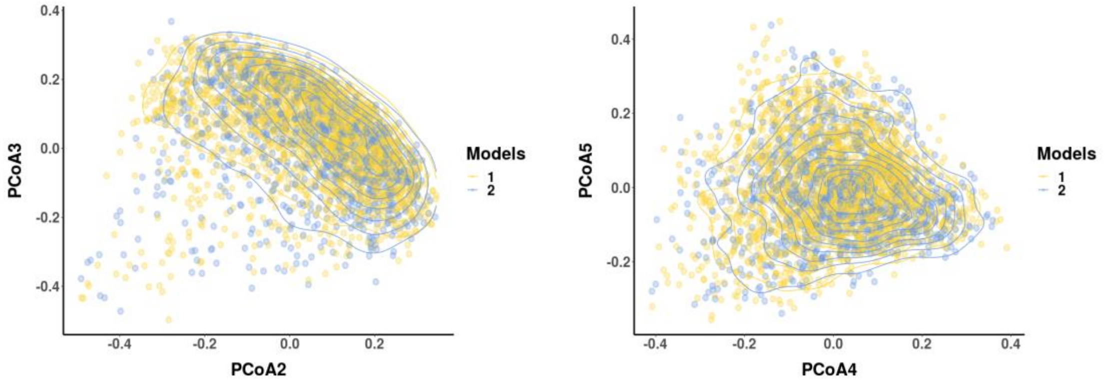

2. The method is promoted as a new method for metagenomic data; but it seems to me that this applies more generally to ecological community profiling data, including 16S and potentially not even limited to microbial ecological communities. Some discussion on the wider scope might be warranted.

Response: As was noted in our response to reviewer #1, we agree with this point - BEEM-Static works for both shotgun metagenomic and 16S data. We have now used “microbiome” instead of “metagenomic” to avoid this confusion.

3. The model is applied for core species that are abundant and prevalent; applicability to lower abundance and rare groups is a possible limitation and could be more clearly stated in Discussion.

Response: We agree with the reviewer and now acknowledge this limitation in the discussion section where we highlight the need for sufficient data to model more species.

4. It would be informative and helpful to show how the term in M-step inside the median (a / \Sigma bx) can be derived for estimating absolute abundances.

Response: We thank the reviewer for this suggestion. We have now added this derivation to the **Supplementary Text**.

5. The "EM framework" is not exactly the classical EM algorithm although very close conceptually; reconsider naming e.g. "iterative EM-type framework" or otherwise acknowledging the difference.

Response: We thank the reviewer for pointing this out. We have now provided the derivation of the EM algorithm for BEEM-Static in the **Supplementary Text**.

6. The bullet point list in 2.7 might be better written out as text or incorporated as a table?

Response: We thank the reviewer for this suggestion and we have now presented these points in the form of a table.

7. principle component analysis (common misspelling) -> should be: principal component analysis; same for principal coordinate analysis

Response: We apologize for this mistake and have now corrected it in the revised version.
